# Hyperparameter Optimization for COVID-19 Pneumonia Diagnosis Based on Chest CT

**DOI:** 10.3390/s21062174

**Published:** 2021-03-20

**Authors:** Paulo Lacerda, Bruno Barros, Célio Albuquerque, Aura Conci

**Affiliations:** Institute of Computing, Fluminense Federal University, Niteroi, RJ 24.310-346, Brazil; brunobarros@id.uff.br (B.B.); celioalbuquerque@id.uff.br (C.A.); aconci@id.uff.br (A.C.)

**Keywords:** computer-aided diagnosis, COVID-19, deep learning, hyperparameter optimization

## Abstract

Convolutional Neural Networks (CNNs) have been successfully applied in the medical diagnosis of different types of diseases. However, selecting the architecture and the best set of hyperparameters among the possible combinations can be a significant challenge. The purpose of this work is to investigate the use of the Hyperband optimization algorithm in the process of optimizing a CNN applied to the diagnosis of SARS-Cov2 disease (COVID-19). The test was performed with the Optuna framework, and the optimization process aimed to optimize four hyperparameters: (1) backbone architecture, (2) the number of inception modules, (3) the number of neurons in the fully connected layers, and (4) the learning rate. CNNs were trained on 2175 computed tomography (CT) images. The CNN that was proposed by the optimization process was a VGG16 with five inception modules, 128 neurons in the two fully connected layers, and a learning rate of 0.0027. The proposed method achieved a sensitivity, precision, and accuracy of 97%, 82%, and 88%, outperforming the sensitivity of the Real-Time Polymerase Chain Reaction (RT-PCR) tests (53–88%) and the accuracy of the diagnosis performed by human experts (72%).

## 1. Introduction

The SARS-Cov2 pandemic has had a worldwide impact [1]. The quantitative Reverse-Transcription Polymerase Chain Reaction (RT-PCR) is considered to be the gold standard for diagnosing COVID-19 [2]. Studies show that initial RT-PCR and chest computed tomography (CT) had comparable diagnostic performance in the identification of suspected COVID-19 patients [3]. To minimize the potential risk of false-negative RT-PCR results, chest CT can be applied for clinically suspected patients with this negative initial result [3]. The research in the area of computer-aided diagnosis for COVID-19 is of great importance. Among the aspects to be investigated, the increment on the efficiency of the health system in detecting and treating the disease is certainly one of them, during and after the pandemic, as well.

Although there are several studies focusing on the use of deep learning to support the diagnosis of COVID-19, as described in Section 1.1, there is still room to improve the sensitivity of the detection of these methods in order to further reduce the number of false negative test results and, consequently, increase the chance of successful detection and treatment of the disease.

The novelty and contribution of this paper are an automatic methodology based on deep transfer learning and hyperparameter optimization techniques on a publicly available dataset of CT images for the computer-aided diagnosis for COVID-19.

### 1.1. Related Works

The use of deep learning in the analysis of medical images, mainly convolutional neural network (CNN) architectures [4], has brought several advances in the area of radiology in recent times [5], efficiently performing tasks, such as diagnosis [6] and segmentation [7]. However, other approaches have also been successful in supporting the diagnosis of lung diseases, such as, for example, neuro-heuristic methods. Authors in [8] use a neuro-heuristic method to build a model that is a fusion of two approaches: a neural network that uses descriptors that are based on the spatial distribution of Hue, Saturation, and Brightness values to pre-select images from the X-ray of sick patients combined with a heuristic search to find the pixels that may represent degenerated tissues, reaching an average accuracy of 79% for the selected dataset. Neuro-fuzzy methods are also used to diagnose lung diseases, as in [9], a work in which the authors developed a pulmonary nodule classifier with 95% accuracy. The proposed classifier contains two sub networks: a fuzzy self organizing network and Multilayer Perceptron (MLP) in a cascaded way. After the lung tomography images go through stages of segmentation and enhancement based on morphological operations, a feature vector is generated to be used as input to the fuzzy layer, which generates a pre-classification vector that is given to MLP for classification.

Some works have already been developed to support the diagnosis of COVID-19 based on tomography images. In [10], authors the propose a method that extracts textural and statistical features from raw CT images and, then, only the best features are selected based on an optimized genetic algorithm. The selected features are serially concatenated and later classified while using the Naive Bayes classifier, achieving 92.6% accuracy and 92.5% sensitivity for SARS-CoV-2 detection based on a public dataset with pneumonia chest CT scans of 35 subjects diagnosed positive of COVID-19. There are also methods based on deep learning [11,12]; however, there are still aspects to be improved. In [13] authors proposed a method based on deep learning to detect COVID-19, community-acquired pneumonia and second pulmonary tuberculosis on computed tomography images. They achieved 95.61% sensitivity for COVID-19 using a two pretrained deep learning models to generate features from computer tomography images, then fuse those features using the discriminant correlation analysis (DCA) method.

### 1.2. Convolutional Neural Networks

VGG represents a family of very deep convolutional networks developed by the Visual Geometry Group at Oxford University. A VGG net is commonly identified by the number of layers that has trainable parameters. For example, the VGG16 [14] presents sixteen layers. It is a network of great depth (16 weight layers) that uses an architecture with very small (3 × 3) convolution filters. VGGs can be considered to be an evolution of classic CNN networks, such as the LeNet-5 [4] and AlexNet [15].

Inception [16] is a CNN architecture that was developed by Google in 2013–2014. It is inspired by an architectural concept, called network-in-network [17]. The unique components of an Inception network are modules that look like small independent networks, which are formed by parallel branches. This structure helps the network learn spatial features separately, which is more efficient than learning them in unity.

ResNet [18] is a network that is based on an element known as residual block. With this element, it was possible to build a network architecture with even more layers (for example, 101 weight layers).

DenseNets [19], or Dense Convolutional Neural Networks, are convolutional neural networks that are composed of dense blocks. This type of architecture was proposed aiming to have fewer parameters and higher accuracy when compared to previous architectures, such as VGGs and Residual Networks [18].

### 1.3. Hyperparameter Optimization

The performance of a neural network is directly related to its training [20], a process in which one seeks to learn the best parameters of the network that minimize a loss function, given a set of sample data and their corresponding labels. However, before the execution of the training process, some parameters must be established, such as the architecture of the neural network, the learning rate, and the optimizer used.

These parameters are known as hyperparameters and they are generally defined by machine-learning engineers based on their empirical experience and intuition [21] developed over time. To make the hyperparameter selection process more efficient, some techniques have been developed over time, such as the Bayesian method [22] and random search [23].

More recently, an optimization method, known as Hyperband [24], has been proposed. This method defines hyperparameter optimization as a pure-exploration non-stochastic infinite-armed bandit (NIAB) problem, a sequential decision problem, where, at each stage, one out of infinite non-stochastic arms is pulled and a reward is obtained according to the chosen sequence.

Hyperband has an approach that seeks to speed up random search through adaptive resource allocation and early-stopping [24]. In the hyperband, each resource corresponds to a hyperparameter to be optimized, such as the neural network architecture, number of layers, or the learning rate allocated to randomly sampled configuration. Hyperband has shown a performance of one order-of-magnitude greater than existing Bayesian optimization methods.

## 2. Materials and Methods

This section describes the method for building a new artificial neural network to support COVID-19 diagnosis based on Convolutional Neural Networks (CNN) [25] and Hyperparameter Optimization [24]. The entire process of building the best diagnostic model can be organized into three stages: data preprocessing, hyperparameters selection, and hyperparameter optimization. At the end of this section, the dataset that was used in the experiments to evaluate the performance of the proposed method is also described.

### 2.1. Data Preprocessing

Each tomographic exam has a specific number of slices. Each slice corresponds to a different position of the tomographic scanner over the patient’s body, which represents a different internal part of the body. In this work, during data preparation (i.e., the preprocessing stage), the 10 more centrally positioned slices of each used exam were selected to standardize the number of slices per scan, concentrating the training of the network on the slices with the greatest potential to present pulmonary area.

When considering the pixel information, the original examination data represented in the DICOM file on Hounsfield units (HU), it is initially quantified as visible image in 256 levels of gray (grades or tones between white and black colors) using the CT lung window. More specifically, a Window Width of 1500 HU and a Window Length of −600 HU are used.

When considering the spatial resolution, or the number of pixels, the original acquired slices are resized to 224 × 224 pixels and normalized between 0 and 1 to adjust them to be used as input to the network architectures that were selected for the hyperparameter optimization.

**Figure 1:**
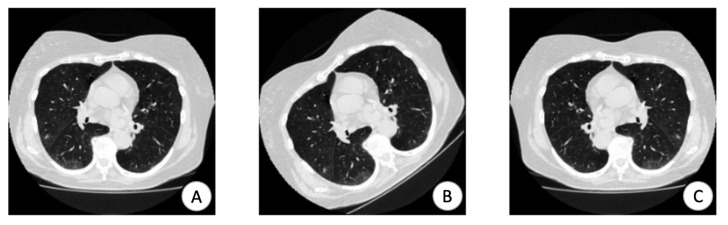
Data augmentation: (**A**) original, (**B**) rotation, and (**C**) flipping.

Moreover, the training data subset is submitted to an augmentation process when fed to the training loop, to increase the size of the dataset and reduce overfitting [26]. In such an augmentation process, each image is randomly flipped horizontally, and randomly rotated in the range [−0.2∗2π,0.2∗2π]. Figure 1 presents an example of the augmentation process that is applied to the training data.

### 2.2. Hyperparameters Selection

Before starting the optimization, it is necessary to select which hyperparameters will be optimized. In this work, the new idea is to build a neural network to support COVID-19 diagnosis by chaining a backbone CNN network for feature extraction, followed by a set of Inception modules, and two fully connected layers. In order to achieve the best training results, four hyperparameters were selected to be optimized in the Hyperparameter Optimization stage: the backbone architecture, the number of Inception modules, the number of neurons in the fully connected layers, and the learning rate.

Four architecture options were selected for the network architecture. They have been published recently (i.e., on the last six years) and they are available in the Keras framework [27]. They are: VGG16 [14], ResNet 101 [18], DenseNet 121 [19], and Inception V3 [28]. All of them have been pre-trained with Imagenet dataset [29].

Table 1 presents the number of backbone parameters of each of the architectures taken as the backbone architecture hyperparameter, in this work.

**Figure 2:**
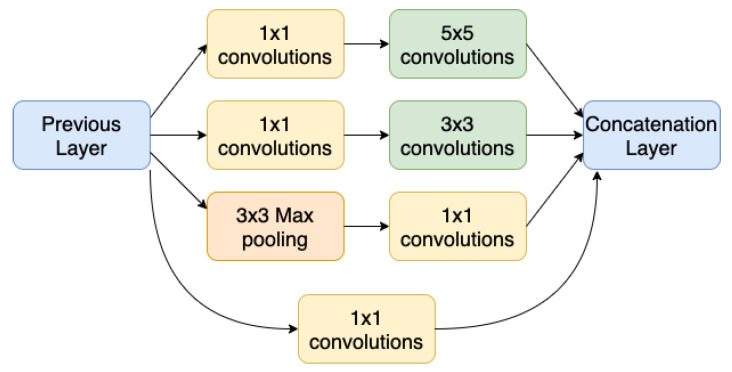
Inception Module.

Although the Inception modules can present some structural variations, their original structure, as introduced by [16] is that illustrated in Figure 2. The inception module is the building block of the Inception network, with which it is possible, instead of choosing a convolution with only one filter size, to concatenate convolution layers with filters of different sizes: 1 × 1, 3 × 3, and 5 × 5. Our proposed architecture will also use inception modules on the top of the backbone based on one of four architectures mentioned before. A typical inception block consists of concatenating the output of a convolution layer with a 1 × 1 filter, with the output of another layer with a 1 × 1 filter followed by a 3 × 3 layer, with the output of another 1 × 1 layer followed by a 5 × 5 layer, and finally a max pooling layer followed by a 1 × 1 convolution. The 1 × 1 layers, also known as Network in Network [17], work as a bottleneck before applying a convolution with a larger filter, such as 3 × 3 or 5 × 5, without increasing too much the computational cost.

Another hyperparameter that was chosen for the optimization stage was the number of hidden units, or neurons, of the two dense or fully connected layers, at the end of the neural network. They come before a third and final dense layer with two units that perform the classification with the softmax activation function. The former two dense layers receive, as input, the features learned by the convolutional layers and improve the extraction of other features to help in the image classification task.

The learning rate is the fourth hyperparameter to be adjusted. It corresponds to the step size, which was used by the optimization algorithm to converge the loss function to a minimum value. A low learning rate can take a long time for the algorithm to converge, while a high learning rate may not lead to the ideal minimum value.

### 2.3. Hyperparameter Optimization Stage

One of the main practical problems experienced in any Deep Learning project is discovering the ideal combination of hyperparameters that minimize or maximize the objective function [30], considering the different topologies of neural networks that are available today. Creating a static search space can be a significant challenge if we consider the large number of parameters combined.

We adopted a framework, called Optuna, to perform the optimization with the Hyperband method and test different combinations of hyperparameters [31]. Optuna is an open-source optimization framework, which allows for us to write complex Deep Learning experiments quickly, efficiently, imperatively, and dynamically.

There are different optimization software, but the following aspects were considered when choosing Optuna: (i) open-source software; (ii) Python language; (iii) dynamic search space; (iv) light enough to run on notebooks—Jupyter Notebook; (v) distributed computing; (vi) web interface (dashboards); and, (vii) implementation of the Hyperband method.

Optuna is organized into several modules, including Study, Storage, Trial, Sampler, and Pruner. The Study module is responsible for managing the value of the objective function for the best set of hyperparameters found, and controlling the optimization method (Hyperband) and number of tests to be performed (Trial).

The objective function and the search space containing the hyperparameters, backbone architecture, the number of inception modules, the number of neurons in the fully connected layers, and the learning rate were previously defined to be used by the Study module.

The Trial module is responsible for monitoring the value of the objective function and the set of hyperparameters managed by the Study module. In addition to this responsibility, the Trial module sends information regarding the hyperparameter sets and the values of the objective function to be stored in the Storage module. This module can store the results in memory or disk, enabling future recovery.

The Sampler module performs the sampling process of the hyperparameters. The Pruner module is responsible for pruning and stopping the tests based on their intermediate values and the previous values that were reported by the Trial module to the Storage module.

In this way, we were able to search for the best hyperparameter configuration, when considering the objective function defined above and the hyperparameters of interest, resulting in the following configurations: VGG16 based convolutional network, five inception modules, 128 neurons in two fully connected layers, and a learning rate of 0.0027.

### 2.4. Dataset

In order to perform the experiments, three public datasets with chest computed tomography (CCT) exams were combined to build the used dataset with 2175 scans, in which 856 present diagnosis of COVID-19 and 1319 are of non-COVID-19 cases. It is worth mentioning that part of the non-COVID-19 samples are not of normal cases, which is they can present CCT with other diseases, such as fibrosis or lung cancer, which are not the focus of this work.

The first dataset is the MosMedData [32], from which 1110 scans were obtained. These exams are from patients distributed in five categories, according to the involvement of the lung area. The first category, with 254 scans, only contains cases that present normal lung tissue with no CT-signs of viral pneumonia. The other four categories, with 856 scans, are cases of COVID-19 with different degrees of severity. In this work, all four categories were labeled as COVID-19 cases.

The second used dataset was obtained from the LUNA16 challenge [33], which contains 888 chest tomography exams with lung nodules. These scans were labeled as non-COVID-19 in the context of this work.

Finally, the third set of data included in our dataset is the one built for the challenge that was promoted by the Open Source Imaging Consortium (OSIC) [34]. This dataset presents 177 chest CT scans from patients with fibrosis. These scans were also labeled as non-COVID-19 in the context of this work.

Table 2 illustrates how the dataset of the testing stage of the proposed method is composed.

The scan slices of the entire dataset are split into three subsets: training, validation, and testing. The division is based on a proportion of approximately 70, 10, and 20 percent, respectively. The result of this division can be seen in Table 3. The data split process was carefully performed to avoid the data leakage problem, in which data from outside the training dataset are used to create the model. In this way, no slice of the training subset belongs to an exam that is present in the test and validation subsets.

Because the total number of scans without COVID-19 is greater than the number with COVID-19, it is necessary to balance these sets to avoid bias to the majority class during training and to use an equitable set also for validation and testing. In the training data, 3380 COVID-19 slices are selected at random and then duplicated to be in the same quantity as the non-COVID-19 slices.

It is significant to note that each duplicated image will go through the data augmentation process before the training, as described in Section 2.1. Thus, during the training phase, the CNN will be fed with randomly augmented images instead of identical copies.

In the validation subset, 390 images of slices of non-COVID-19 scans are discarded at random to match the number of images with COVID-19. With the same goal, 860 slices are removed at random from the test subset.

Table 4 shows the final distribution of the three subsets that were used in the tests and their respective proportions, after oversampling train duplicating some of its samples and undersampling test and validation deleting some of its samples, as described above.

## 3. Results

### 3.1. Baseline Model

A convolutional neural network was trained without hyperparameter optimization to be used as a baseline model in order to better evaluate the benefit obtained by using Hyperband for COVID-19 classification on chest computer tomography. Later, the baseline results will be contrasted to the model trained using the best hyperparameter configuration found by Hyperband.

The network chosen for the baseline is a CNN that uses VGG16 backbone with Imagenet weights for extracting features, a dense layer of 256 neurons at the top, and a learning rate of 0.001. Rather than choosing a simple convolutional network with few layers for the classification task, a VGG16 was chosen, a network that has 16 layers and a backbone, for transfer learning. The VGG16 backbone, the core layers that are responsible for extracting the features from the images, has approximately 14 million parameters.

In short, the baseline network architecture chosen for the backbone, in itself, already produces an excellent performance in the COVID-19 chest CT classification task.

The baseline method produced an accuracy of 87%, and COVID-19 classification sensitivity of 94%. Table 5 presents the results of the classification performed by the baseline model in the test data.

### 3.2. Best Parameter Configuration

The method that was proposed in this article creates an artificial neural network (ANN), while using hyperparameter optimization, presenting a sensitivity of 97%. The proposed ANN architecture reached an accuracy of 88%, COVID-19 sensitivity of 97%, and F1-Score of 89%. It is based on the following four hyperparameters that were selected during the HPO phase: VGG16 [14] base convolutional network, five inception modules [16], 128 neurons in the two fully connected layer, and a learning rate of 0.0027. Table 6 shows a summary of the results that were obtained in the tests using these hyperparameters.

**Figure 3:**
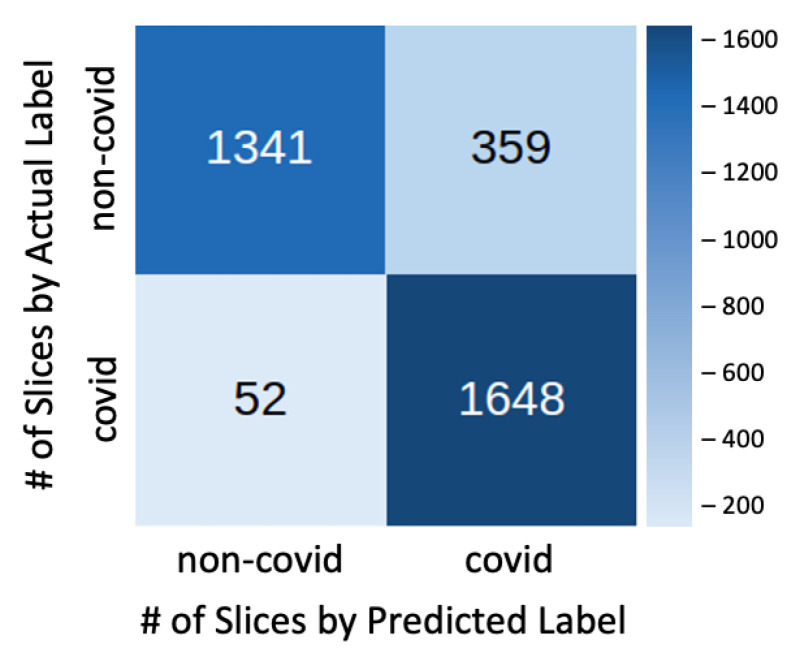
Best Parameters Results Confusion Matrix.

Figure 3 shows the confusion matrix of the test results, where it is possible to analyze the number of true positives, true negatives, false positives, and false negatives.

The Gradient-weighted Class Activation Mapping (Grad-CAM) [35] is a technique that involves superimposing a heatmap over the input images of the convolutional neural network. This heatmap corresponds to a two-dimensional grid with the scores related to a specific class, indicating how important each area of the image is for the classification decision of that class.

**Figure 4:**
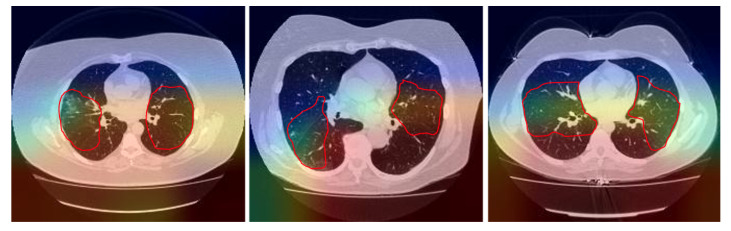
Class Activation Map Visualization.

Figure 4 shows a Grad-CAM visualization for the COVID-19 class with some images from the test dataset. It is possible to notice that the areas with ground-glass opacities and consolidations, typical findings in patients with COVID-19 [36], are highlighted in the heatmap, as indicated by the red outline.

## 4. Discussion

This work proposes a novel method to aid medical doctors in the diagnosis of COVID-19, while using image processing and deep learning techniques, enhanced by hyperparameter optimization [37], applied in the analysis of chest computed tomography (CCT) images.

As stated by [38], the sensitivity of RT-PCR was reported to be modest (53–88%). At the same time, the human-level sensitivity and accuracy of CT are 97%, and 72%, respectively [39].

The network that was designed by the method introduced in this paper achieved 82%, 97%, and 88%, respectively, on precision, sensitivity, and accuracy for the detection of COVID-19. This demonstrated etter sensitivity than RT-PCR, considered the best test for the detection of COVID-19 in the initial phase of the disease, and superior performance to CT analysis undertaken by human specialists in specificity and accuracy. In addition, the proposed use of hyperparameter optimization improved the baseline in all metrics.

The proposed approach is capable of making inferences in real-time, with low cost and better sensitivity and specificity than manual methods. It is mainly based on Convolutional Neural Networks (CNN) to classify CCT images [11].

By analyzing the Confusion Matrix, it is important to highlight the 97% sensitivity in detecting actual COVID-19 cases (1,648 out of 1,700 test cases). Regarding the non-covid-cases, even though 359 cases were misclassified as COVID-19, most of these cases are not normal and they present other diseases, such as fibrosis or lung cancer.

According to the Radiological Society of North America (RSNA) [40], the worldwide shortage of radiologists is a crucial issue. Artificial Intelligence that is applied to medical image analysis plays an essential role in the radiologists’ workflow. The artificial neural network that was proposed by this work can be used by radiologists to support them in the diagnosis of COVID-19, accelerating, and improving this process.

The techniques that were developed by this work can also be adapted and tested to support the diagnosis of other lung diseases, such as tuberculosis and common pneumonia.

The proposed method already uses a smart combination of image processing techniques and neural network architectures. However, other methods involving deep learning, such as extracting features learned by convolutional networks as input to recurrent neural networks [41,42], to take advantage of the temporal characteristic of tomography exams, should be jointly studied and tested to produce a better classification.

The use of the cross-validation technique is important in evaluating machine learning model accuracy. Although it requires higher computational costs, it is subject to less variation, because it uses the entire training set; therefore, in future studies, we will focus on this statistical method to improve the evaluation of our approach.

Additionally, as an evolution of the work, we will consider the evolution of the technique to classify the cases of COVID-19 according to the severity of the case, which is relevant information for the treatment of the disease.

## 5. Conclusions

A computerized technique is proposed in this work for the prediction of SARS-Cov2 disease from the CT scans. The method achieved high accuracy and better sensitivity to COVID-19 (97%) when compared to the performance of human specialists and the sensitivity that was accomplished by the methods presented in other papers.

Based on the performance of the proposed method, it can be used by radiologists to provide a quick and effective diagnosis of the disease, reducing the impact of the high severity cases on the healthcare system.

## Figures and Tables

**Table 1:** Convolutional Neural Network Architectures.

Architecture	Number of Parameters (Backbone)
VGG16	14,714,688
ResNet101	42,658,176
InceptionV3	21,802,784
Densenet121	7,037,504

**Table 2:** Number of computed tomography (CT) Scans by Source dataset.

Class	MosMedData	LUNA16	OSIC	Total
COVID	856	0	0	856
non COVID	254	888	177	1319

**Table 3:** Number of Slices After Training, Validation, and Test Split.

Subset	COVID	non COVID	Total
Train	6020	9400	15,240
Validation	840	1230	2070
Test	1700	2560	4260

**Table 4:** The Number of Slices Used for Training, Validation and Test.

Subset	COVID	non COVID	Percentage
Train	9400	9400	79 %
Validation	840	840	7 %
Test	1700	1700	14 %

**Table 5:** Baseline Results.

Class	Precision	Sensitivity	F1-Score	Accuracy
COVID-19	0.82	0.94	0.88	0.87
Non COVID-19	0.93	0.79	0.86	

**Table 6:** Best Hyperparameters Configuration Results.

Class	Precision	Sensitivity	F1-Score	Accuracy
COVID-19	0.82	0.97	0.89	0.88
Non COVID-19	0.96	0.79	0.87	

## Data Availability

Publicly available datasets were analyzed in this study. MosMed at https://doi.org/10.17816/DD46826; LUNA16 at https://luna16.grand-challenge.org/data; OSIC Challenge at https://www.kaggle.com/c/osic-pulmonary-fibrosis-progression/data.
